# Evaluating the Cytotoxic, Genotoxic, and Toxic Potential of Pyrolytic Tire Char Using Human Lymphocytes and a Bacterial Biosensor

**DOI:** 10.3390/toxics13070582

**Published:** 2025-07-12

**Authors:** Ioanna Efthimiou, Margarita Dormousoglou, Lambrini Giova, Dimitris Vlastos, Stefanos Dailianis, Maria Antonopoulou, Ioannis Konstantinou

**Affiliations:** 1 Department of Biology, School of Natural Sciences, University of Patras, GR-26500 Patras, Greece; lgiova@ac.upatras.gr (L.G.); dvlastos@upatras.gr (D.V.); sdailianis@upatras.gr (S.D.); 2 Department of Sustainable Agriculture, School of Agricultural Sciences, University of Patras, GR-30131 Agrinio, Greece; m.dormousoglou@upatras.gr (M.D.); mantonop@upatras.gr (M.A.); 3 Department of Chemistry, School of Natural Sciences, University of Ioannina, GR-45110 Ioannina, Greece; iokonst@uoi.gr

**Keywords:** pyrolysis, tire char, genotoxicity, toxicity, *Aliivibrio fischeri*, micronuclei

## Abstract

Waste tires (WTs) constitute a potentially significant source of pollution, and the large quantities that are disposed of require proper handling. Pyrolysis has emerged as an environmentally friendly and effective method for WT treatment. In the present study, the cyto-genotoxic and toxic effects of untreated and acid-treated pyrolytic tire char (PTC_UN_ and PTC_AT_, respectively) were investigated. The cytokinesis block micronucleus (CBMN) assay, using human lymphocytes, and the *Aliivibrio fischeri* bioluminescence assay were used for the assessment of cyto-genotoxicity and ecotoxicity, respectively. According to the results, both PTC_UN_ and PTC_AT_ exhibited genotoxicity at all concentrations tested (2.5, 5, and 10 μg mL^−1^), which was more pronounced in the case of PTC_AT_. Cytotoxicity induction was reported for PTC_UN_ and PTC_AT_ at all concentrations. Both demonstrated a relatively low potential for ecotoxicity induction against *A. fischeri*. Since the cyto-genotoxic and toxic effects of PTC_AT_ seemed to be more pronounced, the toxic profile of tire char should be investigated in depth before selecting the appropriate applications, thereby avoiding detrimental effects in the environment and humans alike.

## 1. Introduction

The continuous development of the automobile industry results in the production of large quantities of tires. It is estimated that 1.5 billion tires are sold annually worldwide, while only 50% undergo some sort of processing before being discarded [1]. According to Chen et al. (2021) [2], 13.5 million tons of waste tires (WTs) are produced annually. This number is expected to increase to 1.2 billion by 2030 [3]. To this end, WT handling and processing will constitute a significant problem in both financial and environmental aspects.

Tires consist of a mixture of natural and synthetic rubber. Following their vulcanization, they possess a high carbon content, as well as various organic and inorganic additives with high calorific values [4,5]. Their chemical structure renders their natural decomposition challenging, since their soil decomposition takes approximately 1000 years [2,6,7]. Generally, WTs are discarded in landfills, reclaimed, or incinerated [3,8,9]. WT accumulation could constitute a fire hazard, in addition to being a breeding ground for mosquitoes and bacteria, while improper disposal may pollute both land and water matrices, thus leading to health and environmental hazards [5]. WT incineration produces several harmful byproducts, including polycyclic aromatic hydrocarbons (PAHs), dioxins, and heavy metals [10]. Although WT refurbishment and use are safe and economical management methods, such applications constitute a very small percentage in comparison with the vast production and utilization of WTs. Furthermore, these methods only delay the problem, since WTs will eventually become waste rubber that needs to be processed [11]. As a result, efficient methods of WT processing should be implemented in the framework of environmental protection and the circular economy.

Among the WT processing methods, pyrolysis has proven to be viable for WT valorization. Specifically, pyrolysis is a thermochemical process that involves the thermal decomposition of organic matter in the absence of oxygen. Significant factors that affect the pyrolytic process include temperature, pressure, the catalyst used, and the raw material composition, among others [12,13,14,15,16,17]. This method has been widely used in recent years for waste management, since a significant decrease in waste is accomplished and fewer pollutants are produced [18]. In fact, it constitutes the most efficient and environmentally friendly method for WT treatment and involves the recovery of high-value products, i.e., oil, gas, and carbon black, from the solid char [19]. Notably, the transformation of WTs into alternative fuels leads to the decreased utilization of conventional fossil fuels, and as such, WT pyrolysis could indirectly contribute to the reduction of greenhouse gas emissions [20], thus favoring the reuse of solid char in tire production or their incorporation into electrode materials, activated carbon, and modified asphalt [21,22].

Although WT pyrolysis has been studied quite extensively [23], research regarding the risk posed by pyrolytic tire char (PTC) remains scarce, and further investigation is considered imperative for assessing its environmental and human health effects.

To this end, following the determination of PAHs and major elements of two types of PTC, untreated PTC (PTC_UN_) and acid-treated PTC (PTC_AT_), which were previously chemically characterized [24], were examined in terms of their toxic profiles. In reference to their characterization, PTC_UN_ had a slightly higher content of C, H, N, and S, whereas PTC_AT_ had significantly higher O content. Moreover, the total acidic groups were significantly higher in PTC_AT_. Specifically, the values for carboxylic and phenolic groups were 3.5 and 6 mmol g^−1^ and 37.5 and 7.5 mmol g^−1^ for PTC_UN_ and PTC_AT_, respectively [24]. The toxic and cyto-genotoxic potential of PTC on the bacterium *Aliivibrio fischeri* and human blood peripheral lymphocytes was assessed using the Microtox assay and the Cytokinesis Block Micronucleus (CBMN) assay, respectively. Both assays are well-established and widely used for the assessment of the cytogenotoxic and ecotoxic effects of chemical substances with great precision and reliability [25,26,27]. To the best of our knowledge, the present study is the first to show the risk posed by PTC in human lymphocytes, thus providing new evidence for its utilization and handling/management.

## 2. Materials and Methods

### 2.1. Chemicals and Reagents

Cyclohexane and acetone (ultrapure) were purchased from Pestiscan (Dublin, Ireland). US EPA PAH standard (10 mg/L) in acetonitrile (Supelco, Bellefonte, PA, USA) containing 16 target PAHs (naphthalene, acenaphthylene, acenaphthene, fluorene, phenanthrene, fluoranthene, anthracene, pyrene, chrysene, benzo(a)anthracene, benzo(b)fluoranthene, benzo(k)fluoranthene, benzo(a)pyrene, indeno(1,2,3-c,d)pyrene, dibenzo(a,h)anthracene, and benzo(g,h,i)perylene), was used. Silica gel DSC-Si cartridges (6 mL, Supelco) were used for the clean-up of tire char extracts. HAM’s F-10 medium, L-glutamine, fetal bovine serum (FBS), and phytohaemaglutinin (PHA) were supplied by Gibco (Paisley, Scotland). Giemsa and Mitomycin-C (MMC) were purchased from Sigma-Aldrich Chemical Co. (St. Louis, MO, USA), and Cytochalasin-B (Cyt-B) was purchased from Santa-Cruz (Heidelberg, Germany). All other solvents and chemicals used were of the highest grade commercially available. *A. fischeri* bacteria (Microtox^®^ Acute Reagent) and Microtox^®^ Reconstitution Solution were supplied by Modern Water (York, UK). Sodium chloride, phenol, and ZnSO_4_·7H_2_O were obtained from Sigma-Aldrich (St. Louis, MO, USA). The stocks of the compounds and solutions were kept at 4 °C until use.

### 2.2. PAH Extraction and GC-MS Analysis

For the determination of PAHs, tire char was extracted using the Soxhlet technique according to a previously published method for char [28]. Briefly, 1 g of char was added to the extraction cellulose thimble, and the extraction was performed using 160 mL of an acetone/cyclohexane (*v*/*v*: 1/1) mixture. The extract was filtered and carefully evaporated via vacuum rotary evaporation until it reached a volume of 5 mL and subsequently cleaned via a silica gel cartridge eluted by a 2 × 3 mL acetone/cyclohexane (*v*/*v*: 1/1) mixture, and finally, it was reduced to 1 mL under a gentle stream of nitrogen. An analysis was carried out on a SHIMADZU (Kyoto, Japan) QP-2020 GC–MS system equipped with an AOC-20i autosampler. Separation was achieved using a Rxi–5MS (Restek, Bellefonte, PA, USA) capillary column (30 m × 0.25 mm, 0.25 μm). The ion source temperature was 240 °C, and the injector temperature was 280 °C. Spitless mode was used with a solvent delay time of 2.5 min. Helium was used as the carrier gas, with a constant flow of 1 mL min^−1^. The column oven temperature program started at 50 °C and was increased to 100 °C at 10 °C min^−1^, and then, it was gradually increased to 300 °C at a rate of 5 °C min^−1^, where it was held for 2.5 min. The total program time was 45 min. The injection volume was 1 μL. Ionization was achieved in electron impact mode at 70 eV, and acquisition was performed in single ion monitoring (SIM) mode, setting the corresponding time windows for one quantitative and two qualifier ions for each PAH (Table S1). MS data were obtained in GC–MS operated in full scan mode within the *m*/*z* 50–600 amu mass range. Data were analyzed using GC-MS LabSolutions software (release 4.42).

### 2.3. Determination of Major Elements by X-Ray Fluorescence

Analyses of tire char materials were carried out using Energy-Dispersive X-Ray Fluorescence (EDXRF) spectroscopy equipped with a Canberra SL80175 Si(Li) detector (Canberra Industries, Inc., Meriden, CT, USA).

### 2.4. Sample Preparation

The production of both types of PTC is mentioned in a previous study [24]. Briefly, PTC_UN_ was derived from used rubber tires through pyrolysis (450 °C, oxygen-free atmosphere, under vacuum, 4 h). Afterward, 10 g of PTC_UN_ were suspended in 250 mL of HNO_3_ 2M solution, refluxed for 48 h under vigorous stirring, and washed with distilled H_2_O until the pH reached a value of 7.2 g were suspended in 50 mL of 1-butanol/distilled H_2_O solution (ratio 1:5) and stirred for 12 h, spread in a Petri dish, and dried overnight at 110 °C. The obtained xerogel was calcined at 500 °C under air (1 h, 5 K min^−1^ ramp rate), and PTC_AT_ was the final product. The samples of the untreated and chemically treated PTC were in the form of powder. A stock of 0.2 mg mL^−1^ of each char was prepared in distilled H_2_O and stirred overnight. The concentrations used for the cyto-genotoxic and toxic assessments of both samples were 2.5, 5, and 10 μg mL^−1^. This selection was made after carrying out pilot experiments in order to identify the optimal concentrations according to the criteria established by OECD [27].

### 2.5. Aliivibrio Fischeri Bioluminescence Inhibition Test

The acute toxicity of the PTC was evaluated using the marine luminescent bacteria *A. fischeri*. The analysis was conducted using a Microtox Model 500 Toxicity Analyzer (Azur Environmental, Surrey, UK), according to the 81.9% Basic Test of the Microtox program. Samples in various dilutions were tested in a medium containing 2% NaCl, and luminescence was recorded after 5, 15, and 30 min of incubation at 15 °C. The data were used to calculate the % inhibition of the bacteria’s bioluminescence values using standard MicrotoxOmni Windows Software Version 1.18 (Microtox Omni, York, UK). Each sample was analyzed in triplicate. The experimental procedures and the sensitivity of the *A. fischeri* bacteria were verified via reference positive controls, such as phenol and ZnSO_4_ × 7H_2_O. The effective concentrations obtained (EC_50_) were in accordance with the Microtox^®^ manufacturer’s recommended range, i.e., 21 mg L^−1^ for phenol (5 min) and 5 mg L^−1^ for ZnSO_4_ × 7H_2_O (15 min). Aqueous NaCl solution (2%) was used as a negative reference standard in each measurement. All samples were analyzed in triplicate.

### 2.6. CBMN Assay in Human Lymphocytes In Vitro

#### 2.6.1. Ethics Statement

This research received approval from the Research Ethics Committee (REC) of the University of Patras (UPAT) (Ref. No. 11584/6 March 2018). Blood samples were acquired from two healthy non-smoking male donors (<30 years old), who had not been exposed to radiation, drug treatment, or any viral infection in the recent past.

#### 2.6.2. CBMN Assay Application

The cyto-genotoxic potential of both types of PTC in human lymphocytes was investigated via the in vitro cytokinesis block micronucleus (CBMN) assay using cytochalasin-B (see Supplementary Materials [29,30,31]), according to standard procedures [27]. Mitomycin-C (MMC) was used as a positive control at a concentration of 0.05 μg mL^−1^. The cytokinesis block proliferation index (CBPI) was calculated for the assessment of cytotoxicity by counting 1000 cells for each experimental point, using the following equation:

CBPI = [N1 + N2 + 3(N3 + N4)]/N(1)

where N1, N2, N3, and N4 represent the numbers of cells with one, two, three, and four nuclei, respectively, while N is the total number of cells [32].

### 2.7. Statistical Analysis

Τhe final data are expressed as the mean ± standard deviation (SD) of two independent experiments. Statistical analyses were performed using the SPSS 25 (2019) software package (Armonk, NY: IBM Corp). The inhibition of the bioluminescence (%) (for *A. fischeri)* of each sample, as well as the effective concentration of peroxiredoxin (PRX) that reduces bioluminescence by 50% (EC_50_), were calculated using MicrotoxOmni Windows Software Version 1.18. In the case of human lymphocyte cultures, the significance of the differences between the variables obtained in the control and affected cells was assessed non-parametrically using the Mann–Whitney U test (*p* < 0.05). Datasets were checked for homogeneity of variance (Levene’s test of equality of error variances).

## 3. Results

### 3.1. Determination of PAHs in PTC_UN_ and PTC_AT_

The PAHs determined in both types of PTC, in addition to their concentrations, are presented in Table 1. Regarding PTC_UN_, naphthalene, phenanthrene, anthracene, and benzo(b)fluoranthene were the major PAHs, with values of 312.6, 97.2, 14.9, and 10.7 ng g^−1^, respectively. In the case of PTC_AT_, dibenzo(a,h)anthracene, phenanthrene, naphthalene, and benzo(a)pyrene, with values of 1794.4, 476.2, 281.9, and 40.0 ng g^−1^, respectively, were the PAHs with the highest concentrations. Apart from naphthalene and fluoranthene, all other PAHs possessed higher values in PTC_AT_.

### 3.2. Determination of Major Elements of PTC_UN_ and PTC_AT_

The results of PTC analyses via EDXRF are presented in Table 2, with the major elements being Zn, Fe, Co, and Ca. Except for Co, all other elements have higher values in the case of PTC_AT_.

### 3.3. Toxic Effects of PTC_UN_ and PTC_AT_ on Aliivibrio Fischeri

**Figure 1:**
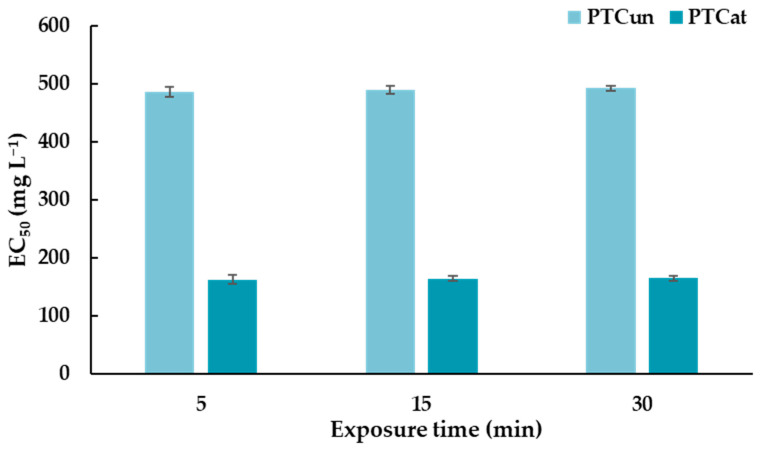
EC_50_ values of untreated and acid-treated PTC at 5, 15, and 30 min of exposure for *Aliivibrio fischeri*.

A Microtox^®^ assay was performed, and the effective concentration of PTC_UN_ and PTC_AT_ that reduced bioluminescence by 50% (EC_50_) was determined after the exposure of *A. fischeri* to different nominal concentrations. Positive (phenol; EC_50_ = 20 mg L^−1^, ZnSO_4_ • 7H_2_O; EC_50_ = 6.5 mg L^−1^, which are within the recommended range of 13–26 mg L^−1^ and 3–10 mg L^−1^, respectively) and negative controls (2% NaCl) were also tested to confirm the accuracy and reliability of the assay. The EC_50_ of PTC_UN_ ranges between 486 and 493 mg L^−1^ (Figure 1) at 5, 15, and 30 min of exposure. In the case of PTC_AT_, the EC_50_ ranges between 163 and 165 mg L^−1^ (Figure 1). The EC_50_ of both types of PTC did not differentiate significantly at 5, 15, and 30 min.

### 3.4. CBMN on Human Lymphocyte Cultures

#### 3.4.1. Genotoxic Effects of PTC_UN_ and PTC_AT_

**Figure 2:**
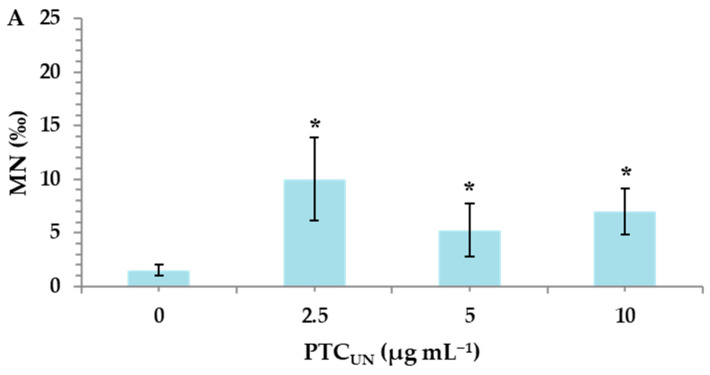

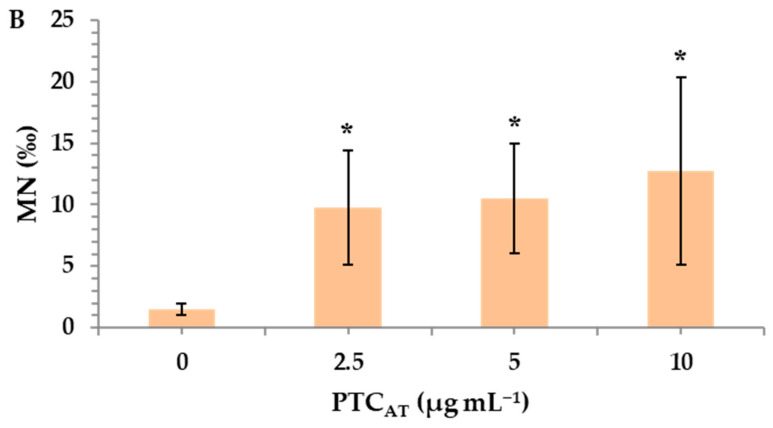
MN frequencies in human lymphocytes treated with 2.5, 5, and 10 μg mL^−1^ of untreated (**A**) and acid-treated (**B**) PTC. Values with an asterisk (*) significantly differ from the control (Mann–Whitney U test, *p* < 0.05).

Untreated and acid-treated PTC were studied at three different nominal concentrations (2.5, 5, and 10 μg mL^−1^) to identify their potential risk of inducing genotoxic effects in cultured human lymphocytes. MMC, a widely used alkylating antibiotic compound with genotoxic potential [33], which was used as a positive control, led to a statistically significant MN induction (26.5 ± 0.7), as expected. A statistically significant difference was observed between the control and both PTC_UN_ and PTC_AT_. Specifically, in PTC_UN_, the highest significant differences in MN frequencies in comparison with the control were recorded in 2.5 μg mL^−1^ (Figure 2A), while in the case of PTC_AT_, a dose-dependent pattern was observed, with all tested concentrations being genotoxic (Figure 2B).

#### 3.4.2. Cytotoxic Effects of PTC_UN_ and PTC_AT_

**Figure 3:**
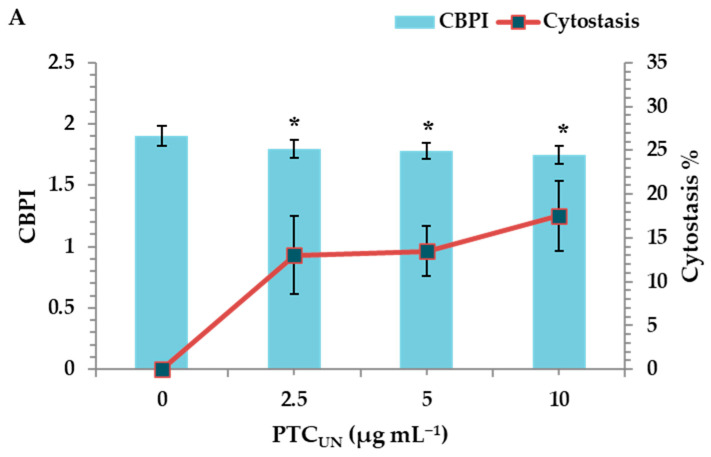

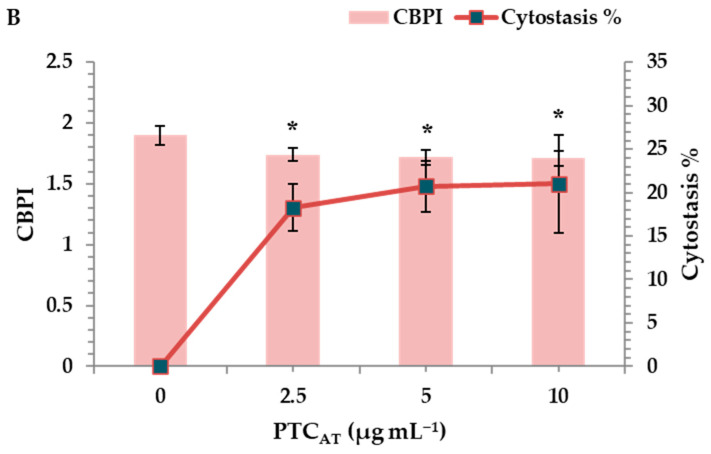
CBPI values and % cytostasis in human lymphocytes treated with 2.5, 5, and 10 μg mL^−1^ of untreated (**A**) and acid-treated (**B**) PTC. Values with an asterisk (*) significantly differ from the control (Mann–Whitney U-test, *p* < 0.05).

The cytotoxic activity of PTC_UN_ and PTC_AT_ was evaluated by determining the cytokinesis block proliferation index (CBPI) and % cytostasis through the CBMN assay. All tested concentrations (2.5, 5, and 10 μg mL^−1^) of PTC_UN_ exhibited cytotoxic activity, with cytostasis ranging from 13% to 17.5% at the highest concentration (Figure 3A). Similarly, PTC_AT_ showed cytotoxic effects across all tested concentrations, with cytostasis ranging from 18.5% to 21% at the highest concentration (Figure 3B).

## 4. Discussion

The overwhelming majority of studies regarding tire char focus on its physicochemical characteristics and potential applications, disregarding its ecotoxicological impact and negative effects on several organisms, including humans. Toxicity studies regarding PTC are very few, since most existing research concerns unprocessed WTs [34]. Thus, after the determination of the major PAHs and elements of two types of PTC, the main focus of the present study was to investigate the toxic and cyto-genotoxic effects of an untreated (PTC_UN_) and acid-treated (PTC_AT_) pyrolytic tire char, providing new information regarding the potential effects of PTC on the environment and humans.

The majority of PAHs detected in PTC exhibited higher concentrations in PTC_AT_. Furthermore, dibenzo(a,h)anthracene and benzo(a)pyrene, which are two of the major PAHs in PTC_AT_, belong in the category of High-Molecular Weight (HMW) PAHs, which are considered more toxic to the environment and its organisms than Low-Molecular Weight (LMW) PAHs, three of which (naphthalene, phenanthrene, and anthracene) constitute major PAHs in the case of PTC_UN_ [35]. As far as the determination of elements is concerned, Zn, which exhibited the highest values in both types of PTC, constitutes one of the most essential elements in car tire production. However, it could also exert toxic effects on different organisms and cell lines [36].

Regarding PTC’s toxicity against *A. fischeri* via the bioluminescence inhibition test, PTC_AT_ and PTC_UN_ had EC_50_ values that ranged between 163 and 165 mg L^−1^ and between 486 and 493 mg L^−1^, respectively. Thus, both types of PTC had relatively high EC_50_ values, demonstrating low potential for ecotoxic effects, while the lower values of PTC_AT_ could suggest a slightly higher risk. Our results are corroborated by similar results presented in a study conducted by Bernardo et al. (2014) [37]. In this study, three types of char were examined for their ecotoxic potential against *A.o fischeri*. The char containing biomass and plastics was not toxic, whereas the other two types of char, which contained tire rubber biomass and tire rubber plastics, significantly inhibited the luminescence of *A. fischeri*. Similarly, tire char from pyrolysis significantly inhibited the growth of algae *Desmodesmus subspicatus,* whereas their toxicity against microcrustacean *Daphnia magna* was negligible, indicating the different responses to the char of different organisms [38].

The present study revealed that both types of PTC were deemed genotoxic, exerting the statistically significant induction of micronuclei (MN) in all tested concentrations (2.5, 5, and 10 μg mL^−1^). However, in the case of PTC_UN_, the lowest concentration (2.5 μg mL^−1^) demonstrated the highest genotoxic potential, whereas PTC_AT_ showed a dose-dependent genotoxic activity. Regarding the former, several compounds, including carbon nanoparticles, form aggregates at higher concentrations, making them less available to cells and unable to enter the cellular membrane [39]. To this end, tire particles were examined for their genotoxic potential by Poma et al. (2019) [40] via the application of a CBMN assay in vitro using the phagocytic cell line RAW 264.7 (mouse leukemic monocyte macrophage cell line). This led to a significant increase in MN frequency in challenged cells only at the lowest dose (10 μg mL^−1^). Furthermore, it should be noted that the cell culture serum contains a variety of proteins, which could create a protein corona around the char surface, creating a barrier between PTC and the cellular membrane [41].

The more pronounced genotoxic activity of PTC_AT_ could be attributed to its physicochemical properties [24]. Due to the acid treatment, more oxidized sites were created on the surface of PTC_AT_. The carboxylic groups were ten-fold higher in PTC_AT_ compared to PTC_UN_ (37.5 mmol g^−1^ and 3.5 mmol g^−1^, respectively) [24]. Carboxylic groups are known to interact with biomolecules, leading to the production of reactive oxygen species (ROS) and causing cellular damage [42]. Moreover, oxidized sites could lead to the increased solubility of char particles and their components, rendering the harmful substances more bioavailable and increasing their toxicity [43]. The highest hydrophilic and aromatic nature of PTC_AT_ was confirmed during its characterization, since the aromatic index ratios H/C and O/C, in addition to the polarity index [(O + N)/C] values, indicate that the polar functional groups on the surface of the PTC_AT_ are significantly increased compared to those in PTC_UN_ [24]. In addition, it has been previously reported that oxidation rates are increased in char with a higher surface area, which is the case in the present study since the surface areas for PTC_UN_ and PTC_AT_ are 20 and 74 m^2^ g^−1^, respectively [24,44].

Regarding the cytotoxicity, although the CBPI values showed a gradient decrease in both cases, the cytotoxic potential was more pronounced in PTC_AT_. The latter could be due to the higher PTC_AT_ surface area and more oxidized sites, as well as its hydrophilic nature that renders compounds more bioavailable, as previously mentioned.

Considering the results regarding the major elements of PTC and the concentrations of the PAHs detected, it is evident that PTC_AT_ could potentially lead to higher ecotoxic effects against *A. fischeri* and exert higher genotoxic and cytotoxic activity against human lymphocytes. In fact, PAHs are known to be mutagenic, leading to potential genetic damage [45]. Benzo(a)pyrene—which was one of the major PAHs in PTC_AT_—is considered one of the most carcinogenic PAHs [46].

Toxic effects exhibited by tire char do not negate its beneficial properties, which include pollutant removal, utilization as construction materials and catalysts, and soil amendment, among others [47]. On the contrary, they facilitate the selection of proper applications according to their physicochemical properties, while at the same time avoiding environmental implications. Thus, PTC_UN_ and PTC_AT_ could potentially be utilized for air purification from pollutants, as construction materials, and as catalysts. Their use in the adsorption of pollutants from aqueous solutions could be considered, with potential applications in waste treatment facilities. However, soil amendment applications should be avoided, since they could negatively impact organisms, including humans, via the food chain. In the same context, Burdová et al. (2025) [38] examined tire char phytotoxicity via a seed germination inhibition test with *Lactuca sativa* var. *capitata.* Although soil microbial activity was ameliorated at 3.5% and 5% *w*/*w* of tire char, the seed germination rate was negatively affected due to the high concentration of PAHs.

The results of the present study demonstrate that both types of PTC induced cyto-genotoxic effects against human lymphocytes, whereas they showed a low chance of ecotoxicity against *A. fischeri*, with PTC_AT_ exhibiting a more toxic profile. Not only does this highlight the fact that future research should focus on the physicochemical characterization and potential applications of PTC but it also demonstrates that it is of the utmost importance that their environmental impact is investigated via the utilization of various model organisms and cell lines. Moreover, different treatments should be applied to the present PTC in order to further attenuate its cyto-genotoxic and toxic potential, which will render it more attractive and improve its status as a candidate for further applications.

## 5. Conclusions

In conclusion, the present study aims to fill a significant gap regarding the assessment of the toxic profile of PTC. It is crucial to evaluate the cyto-genotoxicity and toxicity of tire char using different assays and organisms so that reliable and robust results can be procured. It is emphasized that the toxic profile of char should be thoroughly assessed before utilization, thus avoiding negative environmental effects and health risks. Appropriate applications should be subsequently chosen, taking advantage of the char’s physicochemical characteristics and properties while inhibiting its toxic effects on the environment.

## Figures and Tables

**Table 1:** Concentrations (ng g^−1^) of detected PAHs.

PAH	PTC_UT_ (ng g^−1^)	PTC_AT_ (ng g^−1^)
Naphthalene	312.6	281.9
Acenaphthylene	N.D.	N.D.
Acenaphthene	N.D.	N.D.
Fluorene	N.D.	N.D.
Phenanthrene	97.2	476.2
Fluoranthene	7.5	N.D.
Anthracene	14.9	23.3
Pyrene	8.9	30.1
Chrysene	N.D.	24.8
Benzo(a)anthracene	N.D.	20.2
Benzo(b)fluoranthene	10.7	14.5
Benzo(k)fluoranthene	N.D.	N.D.
Benzo(a)pyrene	6.2	40.0
Indeno(1,2,3-c,d)pyrene	N.D.	N.D.
Dibenzo(a,h)anthracene	7.1	1794.4
Benzo(g,h,i)perylene	N.D.	N.D.

N.D.: Not detected.

**Table 2:** Major elements in PTC_UN_ and PTC_AT_.

Element	PTC_UN_ (% *w*/*w*)	PTC_AT_ (% *w*/*w*)
Zn	2.0 ± 0.4	3.0 ± 0.6
Fe	0.31 ± 0.03	0.37 ± 0.03
Co	0.11 ± 0.02	0.07 ± 0.01
Ca	N.D.	1.0 ± 0.4

N.D.: Not detected.

## Data Availability

Data is contained within the article.

## Supplementary Materials

The following supporting information can be downloaded at: https://www.mdpi.com/article/10.3390/toxics13070582/s1, Table S1: Quantitative and qualifier ions of the target PAHs.

## Abbreviations

The following abbreviations are used in this manuscript:

WT	Waste Tire
PTC	Pyrolytic Tire Char
PTC_UN_	Untreated Pyrolytic Tire Char
PTC_AT_	Acid Treated Pyrolytic Tire Char
PAHs	Polycyclic Aromatic Hydrocarbons
CBMN	Cytokinesis Block MicroNucleus
MN	Micronuclei
CBPI	Cytokinesis-Block Proliferation Index
BN	Binucleated
BNMN	Micronucleated Binucleated Cells
ROS	Reactive Oxygen Species
