# Supplementary material for: Evaluating the Cytotoxic, Genotoxic, and Toxic Potential of Pyrolytic Tire Char Using Human Lymphocytes and a Bacterial Biosensor
Source: Toxics. 2025 Jul 12;13(7):582. doi: 10.3390/toxics13070582 (PMC12298106; doi:10.3390/toxics13070582)
Supplement: Supplementary file 1 [file toxics-13-00582-s001.zip › toxics-3659905-supplementary.pdf]

Supplementary Materials

Evaluating the Cytotoxic, Genotoxic and Toxic Potential of Pyrolytic Tire Char Using Human Lymphocytes and a Bacterial Biosensor

Ioanna Efthimiou [1,2,*], Margarita Dormousoglou [2], Lambrini Giova [1], Dimitris Vlastos [1], Stefanos Dailianis [1], Maria Antonopoulou [2] and Ioannis Konstantinou [3]

[1] Department of Biology, School of Natural Sciences, University of Patras, GR-26500 Patras, Greece
[2] Department of Sustainable Agriculture, School of Agricultural Sciences, University of Patras, GR-30131 Agrinio, Greece
[3] Department of Chemistry, School of Natural Sciences, University of Ioannina, GR-45110 Ioannina, Greece

* Correspondence: iefthimiou@upatras.gr

Table S1. Quantitative and qualifier ions of the target PAHs.

PAH	Quantitative Ion (*m*/*z*)	Qualifier Ions (*m*/*z*)
Naphthalene	128	107, 102
Acenaphthylene	152	150, 153
Acenaphthene	154	152, 153
Fluorene	166	139, 165
Phenanthrene	178	152, 176
Fluoranthene	178	152, 176
Anthracene	202	101, 200
Pyrene	202	101, 200
Chrysene	228	114, 226
Benzo(a)anthracene	228	114, 226
Benzo(b)fluoranthene	252	126, 250
Benzo(k)fluoranthene	252	126, 250
Benzo(a)pyrene	252	126, 250
Indeno(1,2,3-c,d)pyrene	276	138, 277
Dibenzo(a,h)anthracene	278	276, 279
Benzo(g,h,i)perylene	276	138, 277

CBMN assay in human lymphocytes

The whole procedure was performed according to OECD 487 (2023). Specifically,0.5 mL of whole blood from each donor (samples were kept under sterile conditions in heparinized tubes) were added in 6.5 mL Ham's F-10 medium, including 1.5 mL foetal bovine serum and 0.3 mL phytohaemagglutinin, to stimulate cell division. All samples were placed at 37 °C, under 5 % CO_2, for 24 h (Thermo Scientific Incubator) and thereafter appropriate volumes of each stock solution were added, according to the concentration tested. After 20 h of culture treatment with PTCs, cytochalasin-B (Cyt-B, at a final concentration of 6 μg mL^{-1}) was added in any case, for the prevention of cytokinesis (inhibition of actin polymerization), thus favoring nuclear division and the formation of binucleated (BN) cells with low baseline micronuclei frequency (Surrallés et al., 1992). All cultures were incubated for a total period of 72 h. Then, cells were harvested, collected by centrifugation (252 x *g*, 10 min) and treated for 3 min with a mild hypotonic solution (Ham's medium and Milli-Q water, ratio 3:1) at room temperature, followed by fixation with freshly prepared methanol/acetic acid (ratio 5:1, in triplicate). Finally, cells were stained with Giemsa 7% v/v and prepared for further analysis.

CBPI was evaluated by counting at least 1000 cells in any case, following the equation:

$$CBPI = [N1+2N2+3(N3+N4)]/N \quad (1)$$

where N1, N2, N3 and N4 correspond to the numbers of cells with one, two, three and four nuclei and N is the total number of cells (Surrallés et al., 1995).

MN frequency in binucleated (BN) cells was evaluated automatically, using the MNScore slide-scanning platform of the Metafer system (MetaSystems, Altlussheim, Germany). The latter includes a motorized microscope (AxioImager Z1, Carl Zeiss, Jena, Germany) with fluorescence illumination, a motorized X/Y scanning stage (Maerzhaeuser, Wetzlar, Germany) with a range of 225 × 76 mm, a high-resolution monochrome megapixel charge-coupled device (CCD) camera (M4+; JAI AS, Glostrup/Copenhagen, Denmark), and a Windows™ compatible PC (DELL, Langen, Germany) with the Metafer software. At least 2000 BN cells with preserved cytoplasm were scored in any case, following well-established criteria (Fenech, 1997; Fenech et al., 2003).

Additional References

Surrallés, J.; Carbonell, E.; Marcos, R.; Degrassi, F.; Antoccia, A.; Tanzarella, C. A Collaborative Study on the Improvement of the Micronucleus Test in Cultured Human Lymphocytes. *Mutagenesis* **1992**, *7*, 407–410, doi:10.1093/mutage/7.6.407.

Surrallés, J., Xamena, N., Creus, A., Catalan, J., Norppa, H., Marcos, R., 1995. Induction of micronuclei by five pyrethroid insecticides in whole-blood and isolated human lymphocyte cultures. Mutat. Res. 341, 169-184.

Fenech, M. 1997., The advantages and disadvantages of the cytokinesis-block micronucleus method. Mutat. Res. 392, 11-18.

Fenech, M.; Chang, W.P.; Kirsch-Volders, M.; Holland, N.; Bonassi, S.; Zeiger, E.; HUman MicronNucleus project HUMN Project: Detailed Description of the Scoring Criteria for the Cytokinesis-Block Micronucleus Assay Using Isolated Human Lymphocyte Cultures. *Mutat. Res.* **2003**, *534*, 65–75, doi:10.1016/s1383-5718(02)00249-8.
